# “It’s like your days are empty and yet there’s life all around”: A mixed methods, multi-site study exploring boredom during and following homelessness

**DOI:** 10.1371/journal.pone.0302900

**Published:** 2024-05-23

**Authors:** Carrie Anne Marshall, Abrial Cooke, Julia Holmes, Jordana Bengall, Suliman Aryobi, Brooke Phillips, Rosemary Lysaght, Rebecca Gewurtz

**Affiliations:** 1 Social Justice in Mental Health Research Lab, School of Occupational Therapy, Western University, London, Ontario, Canada; 2 School of Rehabilitation Sciences, McMaster University, Hamilton, Ontario, Canada; 3 School of Rehabilitation Science, Queen’s University, Kingston, Ontario, Canada; University of Central Lancashire, UNITED KINGDOM

## Abstract

**Purpose:**

To identify experiences of boredom and associations with psychosocial well-being during and following homelessness.

**Methods:**

Using a convergent, mixed-methods explanatory design, we conducted quantitative interviews with 164 participants) (n = 102 unhoused; n = 62 housed following homelessness) using a 92-item protocol involving demographic components and seven standardized measures of psychosocial well-being. A sub-sample (n = 32) was approached to participate in qualitative interviews. Data were analyzed by group (unhoused; housed). Quantitative data were analyzed using descriptive statistics designed to generate insights into boredom, meaningful activity engagement, and their associations with psychosocial well-being during and following homelessness. Qualitative data were analyzed using thematic analysis. Quantitative and qualitative findings were integrated at the stage of discussion.

**Results:**

Quantitative analyses revealed small to moderate correlations between boredom and increased hopelessness (rs = .376, p < .01), increased drug use (rs = .194, *p* < .05), and lowered mental well-being (rs = -.366, p < .01). There were no statistically significant differences between unhoused and housed participants on any standardized measures. Hierarchical regression analyses revealed that housing status was not a significant predictor of boredom or meaningful activity engagement (*p*>.05). Qualitative interviews revealed profound boredom during and following homelessness imposing negative influences on mental well-being and driving substance use.

**Conclusions:**

Boredom and meaningful activity are important outcomes that require focused attention in services designed to support individuals during and following homelessness. Attention to this construct in future research, practice, and policy has the potential to support the well-being of individuals who experience homelessness, and to contribute to efforts aimed at homelessness prevention.

## Introduction

Identifying and evaluating approaches aimed at supporting individuals to secure and sustain a tenancy has been the focus of much literature on homelessness; however, less attention has been devoted to targeting indices of psychosocial well-being *after* securing a tenancy [1]. Recent research exploring what is needed for thriving following homelessness has highlighted that meaningful activity engagement is a key outcome [1–3]. Persons with experiences of homelessness are frequently excluded from opportunities to engage in activities that are meaningful due to the structural and institutional contexts in which they are embedded [4–7]. Exclusion from meaningful activity can result in deep degrees of boredom that pervade the lives of persons with experiences of homelessness, resulting in lowered mental well-being, profound hopelessness, and questioning one’s very existence [8]. There is an intimate relationship between boredom and meaningful activity engagement in that a lack of activity that is meaningful as defined by the person, can result in the emergence of boredom [4, 5, 8, 10]. To date, research on boredom with persons with experiences of homelessness has been largely exploratory, conducted with small sample sizes, and focused primarily on boredom experienced *during* homelessness [4, 7, 9].

### Boredom and its history in academic discourse

Boredom is defined as “the aversive experience of wanting, but being unable, to engage in satisfying activity” [10] (p. 482), or a lack of challenge or meaning in the activities in which one is engaged [11]. As such, a person can be engaged in an activity that they regard as lacking meaning or may be unoccupied entirely–both of which can result in the emergence of boredom. While meaningful activity entails a description of how a person can use their time, boredom can be viewed as the outcome of whether a person is engaged sufficiently in activities which are experienced as meaningful. Research on this construct indicates that boredom is distinct from apathy, anhedonia or depression [12]. Boredom can be experienced as a *state*, arising from a lack of stimulation in one’s environment, or a *trait* wherein boredom is experienced by virtue of one’s personality across environmental contexts [13]. Historically, it was regarded as a nuisance and form of existential suffering primarily experienced by the upper-classes who were privileged enough to not have to work to generate an income [14, 15]. In recent years, however, researchers have recognized boredom to be a phenomenon that disproportionately affects persons who experience economic oppression [16, 17], including homelessness [7, 8]. This construct has long been of interest to philosophers including Kant [18], Schopenhauer [19], Kierkegaard [20], Nietzsche [21] and Heidegger [22, 23], and has recently garnered interest in the social sciences, particularly psychology and occupational therapy. Growing interest in this construct in recent years has resulted in the development of the interdisciplinary field of ‘Boredom Studies’ which includes philosophers, anthropologists, psychologists, sociologists and occupational therapists [24, 25].

### Boredom and its influence on psychosocial well-being

Interdisciplinary research with a range of populations suggests that boredom can impose both positive and negative influences on psychosocial well-being. Researchers have demonstrated, for instance, that the presence of boredom can elicit curiosity and creativity, which are seen to be facilitative of mental health [26]. Much of this literature, however, suggests that boredom has deleterious effects on well-being. High levels of boredom have been associated with increased engagement in substance use [27–29], low self-reported physical and mental health [30], low motivation [31], and involvement in criminal activity [32, 33]. While research with clinical populations is relatively limited, research with inpatient psychiatric populations suggests that boredom arising from few opportunities to engage in meaningful activity may lead to increased smoking, aggression, abscondment, and poorer mental health [34]. This negative impact is concerning in light of the high levels of boredom reported by persons with experiences of homelessness in existing research combined with the disproportionately high prevalence of mental illness and substance use disorder in this population [35].

### Boredom as it relates to homelessness

While research suggests that some boredom is facilitative of mental well-being [26], the boredom experienced by persons with experiences of homelessness has been reported to be profound and pervasive, imposing negative effects on psychosocial well-being [4–8]. Much of this research, however, has been conducted in the context of exploring other phenomena [8], with only a few empirical studies focusing on boredom as it relates to this population specifically [4–7]. While the findings of these studies provide a glimpse into how boredom is experienced, they are few in number, and have been conducted with small sample sizes [4–7]. Only one study has focused on how boredom is experienced in the transition to housing following homelessness with only two participants [5]. Further, these studies have focused on limited geographic contexts and represent only one city in Canada [4, 5] and one in Romania [6, 7]. There is a need to generate data describing how boredom is experienced, and how it may change in the transition to housing following homelessness with larger sample sizes and in multiple contexts. Such findings will produce more generalizable data that can be used to inform policy and practice.

### The current study

The presence of profound boredom in the lives of persons with experiences of homelessness is a serious social justice and health equity issue [4, 5, 7–9]. More research aimed at understanding this experience, how it emerges, and the ways in which it is associated with indices of psychosocial well-being during and following homelessness, is needed. We conducted this research, guided by the lenses of social justice [36] and health equity [37], and the research question: What is the experience of boredom and its association with indices of psychosocial well-being during and following homelessness?

## Materials and methods

We conducted a convergent, mixed-methods study, defined by Creswell and Plano-Clark as collecting and analyzing both qualitative and quantitative data simultaneously [38]. As such, our research is situated within a pragmatic philosophy [38], informed by the belief that findings generated from both qualitative and quantitative methods are equally valuable for understanding a construct of interest. This study builds on pilot research conducted by our team using a similar protocol [4, 5]. While our original intention was to conduct a longitudinal study, a high degree of attrition in our pilot work at follow-up led to choosing a cross-sectional design for the current study.

### Recruitment

After obtaining ethics approval from Western University, we recruited participants over a one-year period from June 19, 2019 to March 13, 2020. Participants were recruited in shelters, drop-in centres, housing case management, and permanent and transitional housing programs in three cities in Ontario, Canada (Kingston, London, and Hamilton). We recruited by: 1) placing advertisements in the common areas of organizations used by persons with experiences of homelessness that provided contact information for the research team and times during which interviewers would be present to conduct interviews; and 2) by asking staff and leadership in these organizations to provide contact information for the research team to potential interviewees. We recruited participants from two groups: 1) persons who were currently unhoused; and 2) persons housed following homelessness.

### Inclusion and exclusion criteria

Using a combination of convenience, snowball, and purposive sampling methods, we recruited two groups of participants who were over the age of 18: 1) persons who were currently unhoused; and 2) persons housed following homelessness. Unhoused participants were included if they had been unhoused for at least one month in their current episode of homelessness. Housed participants were recruited if they had been housed in market, public, transitional, or permanent supportive housing for less than two years following at least one month of homelessness. We recruited individuals who had a history of being “emergency sheltered” or “unsheltered” using the Canadian Definition of Homelessness [39].

### Procedure

Participants who satisfied inclusion criteria met with an interviewer in a private interview space. Each participant was engaged in an informed consent procedure after which they were assigned a participant number to protect their confidentiality. All participants were engaged in quantitative interviews. From this sample, we purposively recruited a smaller diverse sub-sample to engage in qualitative interviews. The letter of information, informed consent, and all interview questions were read aloud during interviews to overcome the possibility of poor literacy. Participants were provided with a visual stimulus identifying the response options for each standardized measure to limit respondent fatigue. The informed consent and all interview questions were translated into a survey using Qualtrics [40], and responses to all quantitative interview questions were recorded by a member of our research team on a tablet computer during interviews. Participants involved in qualitative interviews were asked to identify a pseudonym to assign to their quotes to protect their confidentiality. A list linking participant identifiers and pseudonyms with participants’ real names was created by our research team and kept separate from participants’ data during analysis as per standard processes established by Western University’s Research Ethics Board. Participants were compensated $20 for participating in quantitative interviews, and $40 for participating in mixed interviews.

### Instruments

#### Quantitative interview

Participants were engaged in a 92-item quantitative interview conducted by one of five members of our research team (CM, RG, AC, JB, JH). Interviews were composed of demographic elements (gender, age, sexual orientation, race/ethnicity, marital status, employment status, income source), housing and health status, and seven standardized measures. Each standardized measure and associated internal consistency for the current study and previous research is detailed in Table 1.

**Table 1:** Description of standardized scales and reliability.

Scale	Description	Internal Consistency (IC) in Previous Research	IC in Present Study
Engagement in Meaningful Activities Survey (EMAS)	12-item inventory of one’s engagement in meaningful activity using a 5-point Likert scale ranging from ‘never’ to ‘always.’ A high score indicates a greater degree of engagement in meaningful activities.	α = 0.89 [73]	α = 0.91
Multidimensional State Boredom Scale-8 (MSBS-8)	8-item scale that identifies ‘state’ boredom using a 7-point Likert scale ranging from ‘strongly disagree’ to ‘strongly agree.’ A high score indicates a greater degree of state boredom	α = 0.91 [70]	α = 0.76
Short Warwick-Edinburgh Mental Well-being Scale (SWEMWBS)	7-item inventory of mental well-being using a 5-point Likert scale ranging from ‘none of the time’ to ‘all of the time.’ A high score indicates a higher degree of mental well-being.	α = 0.91 [72]	α = 0.74
Alcohol Use Disorders Identification Test-10 (AUDIT-10)	10-item inventory using a 3–5-point nominal scale corresponding to an established score related to severity of alcohol use. A high score indicates greater use of alcohol.	α = 0.75–0.97 in previous research [45]	α = 0.92
Drug Abuse Screening Test-10 (DAST-10)	10-item dichotomous scale (YES/NO) that assesses the extent of a person’s substance use. A high score indicates greater degree of drug misuse.	α = 0.86 [46]	α = 0.90
Beck Hopelessness Scale (BHS)	20-item dichotomous scale (TRUE/FALSE) that assesses the degree of hopelessness experienced. A high score indicates a greater degree of hopelessness	α = 0.88 [71]	α = 0.713^1^
Community Integration Scale (CIS)	11-item inventory using both a dichotomous scale (YES/NO) and 5 point-Likert scale to identify the extent of one’s physical and psychological (belonging) integration in his or her community. A higher score indicates a greater degree of physical and psychological integration in one’s community.	α = 0.66 (physical integration subscale); α = 0.68 (psychological integration subscale) [4]	α = 0.60^2^ (physical integration subscale); α = 0.61^2^ (psychological integration subscale)

^1^Item 13 of the BHS (“When I look ahead to the future, I expect that I will be happier than I am now”) was removed to improve the reliability of the overall scale. All statistics using the BHS in this study were calculated using this revised scale

^2^In the ‘questionable’ range according to George & Mallery [69]

#### Qualitative interview

Following quantitative interviews, a purposive sub-sample of participants were engaged in semi-structured qualitative interviews aimed at identifying experiences of boredom during and following homelessness. Participants in this sub-sample were selected to obtain a diverse sample based on age, gender, housing status and geographic location. A list of demographic characteristics of qualitative participants was completed after each qualitative interview, and new participants were approached based on the developing composition of the sample to maximize the diversity of qualitative participants. Interviews were recorded on a digital recording device and transcribed verbatim. Qualitative interview questions posed to participants are provided in Table 2.

**Table 2:** Semi-structured qualitative interview questions.

1. Tell me about the activities that are meaningful to you. 2. What do you think allows/prevents you to be/not be involved in these activities? 3. In what ways is it important for you to be involved in activities that are personally meaningful to you, if at all? 4. In what ways do the activities that you spend your time doing relate to having a sense of meaning in your life, if at all? 5. Do you experience boredom throughout the day? a. If so, what does it feel like for you? b. If you experience boredom, tell me why you think it comes up for you. c. If you don’t experience boredom, why do you think that is? 6. Is there anything about you, the way you think, or your health that makes it more or less likely that you’d experience boredom? 7. Is there anything about your environment that you think makes it more or less likely that you’d experience boredom? 8. What are the consequences of experiencing boredom for you, if any? a. How does boredom affect your mental health and well-being, if at all? b. How does boredom affect your physical health and well-being, if at all? 9. What strategies do you use to cope with any boredom that you experience? 10. If you don’t experience much boredom, what strategies are you using now that are helping to reduce boredom, if any? 11. What do you think could be done to reduce any boredom that you experience other than your own personal coping strategies? 12. Is there anything that we haven’t discussed in this interview that is important to mention with respect to how you spend your time and your health and well-being right now?

### Analysis

#### Quantitative

Using SPSS 28, we calculated descriptive statistics for all variables. Demographic, housing and health characteristics were calculated for both unhoused and housed participants and for the full sample. Summary scores were generated for each standardized measure using processes described by the test authors. As we planned to identify any differences on standardized measures between unhoused and housed groups, we conducted analyses to determine any significant differences in demographic, housing or health characteristics that would explain these relationships using Mann-Whitney U, Chi-Square, and Fisher’s Exact Tests as indicated.

Our quantitative analyses were informed by four research sub-objectives, with associated statistical analyses. We opted to use non-parametric tests when possible, given that several sub-tests used ordinal scales. We conducted: 1) Spearman correlations to identify *any statistically significant associations between boredom (MSBS-8) and meaningful activity engagement (EMAS) with indices of psychosocial well-being*; 2) one-sample Wilcoxon Signed-Rank tests to identify *how participants’ scores on standardized measures differed from norms and threshold scores reported in existing literature*; 3) Mann-Whitney U tests to determine *whether boredom*, *meaningful activity*, *and indices of psychosocial well-being were significantly different for participants who were unhoused or housed following homelessness*; and 4) two hierarchical multiple regression analyses to determine *whether months housed or unhoused (in the past year) predicted boredom (MSBS-8) and meaningful activity engagement (EMAS)*, while controlling for the effects of age, gender, and recruitment site. Assumptions of normality, linearity, multicollinearity and homoscedasticity were assessed prior to conducting our analyses and were determined not to have been violated. When conducting these regression statistics, the same independent variables were entered for both analyses. In block one, we entered age, gender and recruitment site. In block two, we entered months housed, and months unhoused in the past year. The dependent variable for the first analysis was total MSBS-8 score, and total EMAS score for the second. During analysis, when data was missing in full or in part in a participant’s response on a specific standardized test, we eliminated that participant from the analysis involving that test. Significance was set to *p* < .05 for all statistical tests.

#### Qualitative

Transcripts were separated by group (unhoused; housed following homelessness) and uploaded to Dedoose [41], a qualitative data management program, to facilitate cross-site collaboration and analysis. Transcripts were coded abductively, informed by the lenses of social justice and health equity, by several members of our research team (CM, AC, JB, JH, SA, BP, RG). Using thematic analysis [42], our team met on several occasions to arrange codes into themes. Following recommendations of Braun & Clarke [42], we identified an overarching essence describing participant narratives from both groups, and also an essence that captured experiences of boredom for both unhoused and housed participants.

***Trustworthiness*.** Trustworthiness was established using criteria described by Lincoln & Guba [43] including: 1) prolonged engagement with the population of interest, established by our research team’s extensive involvement in research and practice related to homelessness; 2) peer debriefing among our research team during the conduct of interviews, and in the process of analyzing our data; 3) recording interviews; 4) accurate transcription; 5) intercoder consensus (see analysis); and 6) use of a computer program to organize qualitative data.

***Reflexivity*.** Collectively, the principal investigator and all members of our research team have decades of experience in research and practice with individuals who experience mental illness, substance use disorders and homelessness. Our extensive involvement in research and practice in this area has informed the design of this study, and how we’ve analyzed our qualitative data. We have embraced this knowledge and believe that these background experiences have enabled us to analyze the narratives of participants with greater depth and sensitivity.

## Results

### Participants

Our full sample consisted of 164 participants, of which 102 were unhoused, and 62 were housed. We intended to recruit similar sample sizes for both groups; however, the COVID-19 pandemic interrupted our recruitment efforts. At the time, our team determined that public health regulations imposed early in the pandemic would significantly influence our findings given our focus on boredom and meaningful activity engagement, and we made the difficult decision to cease recruitment to avoid cohort effects. As such, none of the participants in the current study were interviewed during or after physical distancing restrictions were imposed during the COVID-19 pandemic in the province of recruitment. The demographic characteristics of participants in our sample are provided in Table 3, and the housing and health characteristics of our sample are provided in Table 4.

**Table 3:** Participant demographic characteristics by group (n = 164).

Demographic Characteristics				
	Unhoused (n = 102) n (%)	Housed (n = 62) n (%)	Full Sample (n = 164) n (%)	*P*-value
Gender				.257^4^
Men	57 (55.9)	29 (46.8)	86 (52.4)	
Women	45 (44.1)	32 (51.6)	77 (47.0)	
Other	-	1 (1.6)	1 (0.6)	
Age	(18–82; Mdn = 43.5; IQR = 18.25)	(18–87; Mdn = 46.5; IQR = 19.25)	(18–87; Mdn = 45; IQR = 18.75)	.217^5^
Sexual orientation				.193^6^
Heterosexual	88 (86.3)	46 (74.2)	134 (81.7)	
2SLGBTQ+	14 (13.7)	13 (21.0)	27 (16.5)	
Prefer not to answer	-	3 (4.8)	3 (1.8)	
Race/Ethnicity				.973^6^
White	80 (78.4)	51 (82.3)	131 (78.9)	
First Nations	11 (10.8)^1^	8 (12.9)^2^	19 (11.6)	
Black	3 (2.9)	2 (3.2)	5 (3.0)	
Metis	2 (2.0)	1 (1.6)	3 (1.8)	
Hispanic	2 (2.0)	-	2 (1.2)	
Mixed Race	3 (2.9)	2 (3.2)^3^	5 (3.0)	
Prefer not to answer	1 (1.0)	-	1 (0.6)	
Marital status				**.015** ^**6**^ *
Single	63 (61.8)	27 (43.5)	90 (54.9)	
Divorced/Separated	15 (14.7)	19 (30.6)	34 (20.7)	
Married/Common-law	12 (11.8)	12 (19.4)	24 (14.6)	
Widowed	9 (8.8)	2 (3.2)	11 (6.7)	
Prefer not to answer	3 (2.9)	2 (3.2)	5 (3.0)	
Employment status				.122^4^
Employed	12 (11.8)	13 (21.0)	25 (15.2)	
Permanent full-time	1 (1.0)	3 (4.8)	4 (2.4)	
Permanent part-time	3 (2.9)	4 (6.5)	7 (4.3)	
Self-employed	4 (3.9)	1 (1.6)	5 (3.0)	
Contract/relief/temporary	4 (3.9)	5 (8.1)	9 (5.5)	
Unemployed	90 (88.2)	49 (79.0)	139 (84.8)	
Income source^7^				
ODSP	48 (47.1)	42 (67.7)	90 (54.9)	**0.010** ^**4**^ *
OW	37 (36.3)	12 (19.4)	49 (29.9)	**0.022** ^**4**^ *
Employment	12 (11.8)	13 (21.0)	25 (15.2)	
OAS	10 (9.8)	7 (11.3)	17 (10.4)	
Disability pension	3 (2.9)	2 (3.2)	5 (3.0)	
Panhandling	3 (2.9)	3 (4.8)	6 (3.4)	
Selling drugs	3 (2.9)	-	3 (1.8)	
Family/friends	-	2 (3.2)	2 (1.2)	
EI	1 (1.0)	-	1 (0.6)	
CCTB	1 (1.0)	-	1 (0.6)	
Participation in research studies	1 (1.0)	-	1 (0.6)	
Survivor’s benefit	1 (1.0)	-	1 (0.6)	
Inheritance	1 (1.0)	-	1 (0.6)	
Honoraria from volunteering	-	1 (1.6)	1 (0.6)	

**Note:** Percentages do not all equal 100 due to rounding

^1^Cree = 1; Lower Cayuga = 1; M’Chigeeng = 1; Mohawk = 2; Ojibway = 2; Other = 2; Unspecified = 2

^2^Cree = 1; Mohawk = 1; Mohawk/Ojibway = 1; Oneida = 1; Other = 2; Unspecified = 2

^3^Two participants in this group identified as both mixed race and First Nations, leading to a discrepancy in the frequencies reported

^4^Chi-Square Test of Independence

^5^Mann-Witney U Test (2-sided)

^6^Fisher’s Exact Test (used due to the presences of frequencies <5 in one or more cells)

^7^Due to multiple possible income sources, differences between participant groups on income source was only calculated for social assistance given that these categories were mutually exclusive

ODSP = Ontario Disability Support Program (disability-related social assistance); OW = Ontario Works (general social assistance); OAS = Old Age Security; EI = Employment Insurance; CCTB = Canada Child Tax Benefit

*p < .05

**Table 4:** Participant housing and health status by group (n = 164).

Housing/Health Status				
	Unhoused (n = 102) n (%)	Housed (n = 62) n (%)	Full Sample (n = 164) n (%)	*P*-value
Housing History				
Months in current housing	-	(1–21; Mdn = 7.5; IQR = 7)	-	-
Where are you housed?	-		-	-
Market rent apartment	-	21 (33.9)	-	-
Transitional housing	-	14 (22.6)	-	-
Rent geared to income housing	-	12 (19.4)	-	-
Permanent supportive housing (cluster site)	-	8 (12.9)	-	-
Permanent supportive housing (scatter site)	-	3 (4.8)	-	-
Other	-	4 (6.5)	-	-
How many times have you lost your housing in the past three years?	(4–7; Mdn = 4; IQR = 2)	(4–7; Mdn = 4; IQR = 1)	(4–7; Mdn = 4; IQR = 1)	.138^2^
Months unhoused in the past year	(1–12; Mdn = 8.5; IQR = 8)	(0–12; Mdn = 3; IQR = 6)	(0–12; Mdn = 6; IQR = 9)	-
Months unhoused (lifetime)	(1–300; Mdn = 24; IQR = 50)	(3–240; Mdn = 24; IQR = 39.5)	(1–300; Mdn = 24; IQR = 38)	.694^2^
Unhoused sleep location				
Shelters	97 (95.1)	-	-	-
Outdoors	46 (45.1)	-	-	-
Couch-surfing/with friends	30 (29.4)	-	-	-
Other	21 (20.6)	-	-	-
Health Status				
Physical health conditions				
MSK	28 (27.5)	26 (41.9)	54 (32.9)	.062^3^
Respiratory	20 (19.6)	5 (8.1)	25 (15.4)	*.046 ^3^
Digestive	11 (10.8)	3 (4.8)	14 (8.5)	.186^3^
Diabetes	11 (10.8)	-	11 (6.7)	*.007 ^4^
Infectious diseases	8 (7.9)	7 (11.3)	15 (9.1)	.458^3^
Cardiovascular	9 (8.8)	5 (8.1)	14 (8.5)	.866^3^
Skin	4 (3.9)	1 (1.6)	5 (3.0)	.651^4^
Cancer	2 (2.0)	-	2 (1.2)	.527^4^
Other	19 (18.6)	9 (14.5)	28 (17.1)	-
Cognitive conditions				
Attention (e.g. ADD/ADHD)	14 (13.7)	8 (12.9)	22 (13.4)	.451^3^
Brain injury	11 (10.8)	6 (9.7)	17 (10.4)	.822^3^
Learning disabilities (e.g. dyslexia)	5 (4.9)	4 (6.5)	9 (5.5)	.883^3^
Epilepsy	1 (1.0)	-	1 (0.6)	-
Mental health conditions				
Mood disorders	46 (45.1)	25 (40.3)	71 (43.3)	.765^3^
Anxiety disorder	32 (31.4)	24 (38.7)	56 (34.1)	.534^3^
Psychotic disorder	18 (17.6)	9 (14.5)	27 (16.5)	.600^3^
Substance use disorder	15 (14.7)	7 (11.3)	22 (13.4)	.534^3^
Personality disorder	11 (10.8)	8 (12.9)	19 (11.6)	.920^3^
Obsessive compulsive and related disorders	7 (6.9)	6 (9.7)	13 (7.9)	.559^4^
Stress and trauma-related disorders	24 (23.5)	18 (29.0)	42 (25.6)	.434^3^

**Note:** Percentages do not all equal 100 due to rounding

^1^Cumulative frequencies in this category exceeds the group participant size as participants used multiple sleep locations

^2^Mann-Witney U Test (2-sided)

^3^Chi-Square Test of Independence

^4^Fisher’s Exact Test (used because frequencies <5 in one or more cells)

Housed and unhoused participants were mostly similar on demographic characteristics, with significant differences in marital status (*p* < .05) and type of social assistance received (*p* < .01; *p* < .05) (see Table 2). There were no statistically significant differences between groups in terms of housing history other than participants’ current housing status. With respect to health, the groups were mostly similar with a significantly greater number of participants living with respiratory conditions (*p* < .05) and diabetes (*p* < .01) in the unhoused group (see Table 3).

### Quantitative findings

#### Associations between boredom, meaningful activity, and indices of psychosocial well-being

A statistically significant negative correlation between boredom and meaningful activity was observed (*rs* = -.222, *p* < .01). Using criteria established by Cohen [44], the strength of this correlation was small. Higher EMAS scores were significantly correlated with increased physical community integration (*rs* = .237, *p* < .01, small), decreased drug use (*rs* = -.289, *p* < .01, small), and increased mental well-being (*rs* = .468, *p* < .01, moderate). Greater reported boredom was associated with increased reported hopelessness (*rs* = .376, *p* < .01, moderate), increased drug use (*rs* = .194, p < .05, small), and decreased mental well-being (*rs* = -.366, *p* < .01, moderate). No statistically significant correlations were reported between boredom and meaningful activity engagement with psychological community integration or alcohol use. See Table 5.

**Table 5:** Correlations between EMAS & MSBS and measures of psychosocial wellbeing (n = 164).

Scale	MSBS-8	BHS	CIS-Phys	CIS-Psyc	AUDIT-10	DAST-10	SWEMWBS
**Meaningful Activity Engagement (EMAS)**	-.222**	.135	.237**	.037	-.041	-.289**	.468**
**Boredom (MSBS-8)**	-	.376**	-.072	-.024	-.047	.194*	-.366**

*p < .05 (two-tailed

**p < .01 (two-tailed)

Note. EMAS = Engagement in Meaningful Activities Survey; MSBS-8 = Multidimensional State Boredom Scale– 8-item version; CIS-Phys = Community Integration Scale, Physical Integration; CIS-Psyc = Community Integration Scale, Psychological Integration; AUDIT-10 = Alcohol Use Disorders Identification Test– 10-item version; DAST-10 = Drug Abuse Screening Test– 10-item version; SWEMWBS = Short Warwick-Edinburgh Mental Well-Being Scale.

#### Differences between boredom, meaningful activity engagement and indices of psychosocial well-being from published norms and threshold scores

Norms and threshold scores were available only for measures of boredom (MSBS-8), meaningful activity (EMAS), mental well-being (SWEMWBS, BHS), and substance use (AUDIT-10, DAST-10). Significant differences across all measures were observed. Compared with norms reported in existing literature, participants in our study reported: significantly higher boredom on the MSBS (*z* = 9.909, *p* < .0001); significantly lower engagement in meaningful activity on the EMAS (*z* = -6,765, *p* < .0001); lower mental well-being on the SWEMWBS (*z* = -7.029, *p* < .0001); and higher degrees of hopelessness on the BHS (*z* = -8.361, *p* < .0001). When compared with threshold scores established by the test authors for substance use, participants in our study were engaged in alcohol use that was significantly lower than a ‘hazardous’ threshold score on the AUDIT-10 [45], and significantly lower than a ‘low-moderate’ range on the DAST-10 [46]. All of these differences were associated with moderate to large effect sizes. See Table 6.

**Table 6:** One-sample wilcoxan signed rank tests for indices of boredom, meaningful activity and psychosocial well-being.

Construct	*n*	Mdn (IQR)	Comparison Value	Z	*p* (2-tailed)	Effect Size (r)
Meaningful Activity						
EMAS	163	43 (15)	48.2^a^	-6.765	.000***	.53^b^
Boredom						
MSBS	163	40 (12)	27.84^c^	9.909	.000***	.78^b^
Mental Well-Being						
SWEMWBS	159	20.73 (4.62)	23.2^d^	-7.029	.000***	.56^b^
BHS	162	10 (4)^i^	8.36^e^	8.361	.000***	.66^b^
Substance Use						
AUDIT-10	164	2 (6)	8.0^f^	-5.469	.001***	.43^g^
DAST-10	164	3 (7)	5.0^h^	-5.131	.001***	.40^g^

Note. Mdn = Median; IQR = Interquartile range

Note. EMAS = Engagement in Meaningful Activities Survey; MSBS-8 = Multidimensional State Boredom Scale– 8-item version; CIS-Phys = Community Integration Scale, Physical Integration; CIS-Psyc = Community Integration Scale, Psychological Integration; AUDIT-10 = Alcohol Use Disorders Identification Test– 10-item version; DAST-10 = Drug Abuse Screening Test– 10-item version; SWEMWBS = Short Warwick-Edinburgh Mental Well-Being Scale.

^a^ Mean derived from a psychometric study evaluating the EMAS with n = 154 older adults [75]

^b^ Corresponds to a ‘large’ effect size according to Sullivan & Feinn [76]

^c^ Mean derived from a large population sample of the general population in Australia [77]

^d^ Mean derived from a large population sample of the general population in the UK, 2011 [78]

^e^ Mean derived from a study on the psychometric properties of the BHS in a sample of n = 411 outpatients [74]

^f^ Threshold score derived from Reinert & Allen (2007) identifying a score of 8 or higher as ‘hazardous drinking’

^g^ Corresponds to a ‘medium’ effect size according to Sullivan & Feinn [76]

^h^ Threshold score derived from Cocco & Carey [79] identifying scores of 5 or lower as ‘low-moderate’ drug use

^i^ While we have used a revised scale for other analyses, we have used participant ratings for the full BHS for this analysis given that our comparison value was calculated based on the full measure

***p < .001

#### Differences between boredom, meaningful activity, and indices of psychosocial well-being during and following homelessness

There were no statistically significant differences on measures of boredom (MSBS-8), meaningful activity (EMAS), mental well-being (SWEMWBS, BHS), substance use (AUDIT-10, DAST-10), and community integration (physical integration, psychological integration) between unhoused and housed groups. Persons who were housed reported higher levels of psychological integration in their communities, however, this finding only *approached* statistical significance [Unhoused (Mdn = 10, n = 98); Housed (Mdn = 11.5, n = 62), *U* = 2502, z = -1.895, p = .058, r = .15)]. See Table 7.

**Table 7:** Mann Whitney U tests comparing housed and unhoused participants on indices of psychosocial well-being (n = 164).

Construct								
	Unhoused		Housed		U	Z-Score	*P*-value	*Effect size* *(r)*
	*n* ^1^	Mdn (IQR)	*n* ^2^	Mdn (IQR)				
Meaningful Activity								
EMAS	101	44 (14.5)	62	42.5 (17.75)	3073	-.200	.841	.02
Boredom								
MSBS	101	41 (12)	62	38.5 (13.75)	2892	-.818	.414	.06
Mental Well-Being								
SWEMWBS	98	20.73 (4.62)	61	19.98 (4.62)	2782	-.737	.461	.06
BHS	100	7 (4)	62	6 (4)	2934	-.576	.564	.05
Substance Use								
AUDIT-10	102	3 (7)	62	2 (6)	3089	-.253	.800	.02
DAST-10	102	4 (6)	62	3 (7)	2863	-1.027	.304	.08
Community Integration								
Physical Integration	97	3 (3)	61	3 (3)	2949	-.033	.974	.00
Psychological Integration	98	10 (4)	62	11.5 (3)	2502	-1.895	.058	.15

Note. EMAS = Engagement in Meaningful Activities Survey; MSBS-8 = Multidimensional State Boredom Scale– 8-item version; CIS-Phys = Community Integration Scale, Physical Integration; CIS-Psyc = Community Integration Scale, Psychological Integration; AUDIT-10 = Alcohol Use Disorders Identification Test– 10-item version; DAST-10 = Drug Abuse Screening Test– 10-item version; SWEMWBS = Short Warwick-Edinburgh Mental Well-Being Scale.

^1^Incomplete data for some participants meant that summary scores could not be generated for some participants, thereby reducing the overall sample size for associated standardized measures

#### Housing status and associations with boredom and meaningful activity engagement

We conducted two hierarchical regression analyses. In the first analysis, we sought to identify how boredom (measured by total MSBS-8 score) was predicted by housing status (months unhoused/months housed in the past year) while controlling for the effects of age, gender, and recruitment site. This model was found to be non-significant (*p*>.05). Age, gender, and site were entered in block one, explaining 6% of the variance in MSBS-8 scores. After entering months housed and months unhoused (in the past year) in block two, the total variance explained by the model as a whole was 6.4%, *F*(6, 149) = 1.7, *p* = .125. The two independent variables (months housed and months unhoused) explained an additional 0.4% of the variance in MSBS-8 scores, *R* squared change = .004, *F* change (2, 149) = .311, *p* = .733. In the final model, only one recruitment site (London) was statistically significant (beta = .20, *p* < .05).

In the second analysis, we sought to identify how engagement in meaningful activity (measured by the EMAS) was predicted by housing status (months unhoused/months housed in the past year) while controlling for the effects of age, gender and recruitment site. This model was found to be non-significant (*p*>.05). Age, gender, and site were entered in block one, explaining 1.2% of the variance in EMAS scores. After entering months housed and months unhoused (in the past year) into block two, the total variance explained by the model as a whole was 2%, *F*(6, 149) = .505. The two independent variables (months housed and months unhoused) explained an additional 0.8% of the variance in EMAS scores, *R* squared change = .008, *F* change (2, 149) = .636. In the final model, none of the independent variables were significantly associated with EMAS scores.

### Qualitative findings

#### Participants

From the larger sample, we interviewed 32 participants using our qualitative interview protocol (n = 18 unhoused; n = 14 housed). Participants in the unhoused group included nine women (50%) and nine men (50%) ranging in age from 18–56 (Mdn = 38; IQR = 11). Participants in the housed group included six women (43%) and eight men (57%) ranging in age from 23–77 (Mdn = 39; IQR = 28). None of the participants in these two groups identified as non-binary or with other genders.

#### Overarching essence—Boredom is “like being caught in a tornado.”

The essence of our analysis across both housed and unhoused groups represented the feeling of boredom described by participants, which permeated much of their days during and following homelessness. During homelessness, participants described how their time use was restricted to activities that enabled survival, and the rules and hours of operation of the programs that they accessed. These influences restricted their time use and resulted in long periods where time was relatively unoccupied: “*There’s too much time on my hands*, *here*” [Serina, Unhoused]. Following homelessness, participants’ time use was determined by finances, the presence of social relationships, and stigma. Restrictions imposed by these factors led to experiencing boredom “*almost every moment of the day*” [Andrew, Housed]. Both during and following homelessness, participants felt trapped in the deep degrees of boredom that they experienced. One participant described the feeling of relentless boredom in his life as “*like being caught in a tornado*” [Shawn, Unhoused]. This metaphor describes the calm in the centre of a tornado, surrounded by chaos and fast-moving wind. Participants described feeling like they were caught in a tornado by being embedded in a society that they could not access and in which they had few opportunities to participate. The environments in which they were embedded severely limited opportunities for participating in activities that were meaningful resulting in a feeling of unwelcome stillness. This feeling persisted while movement, people and opportunities surrounded them that they could only observe as an outsider.

**Fig 1:**
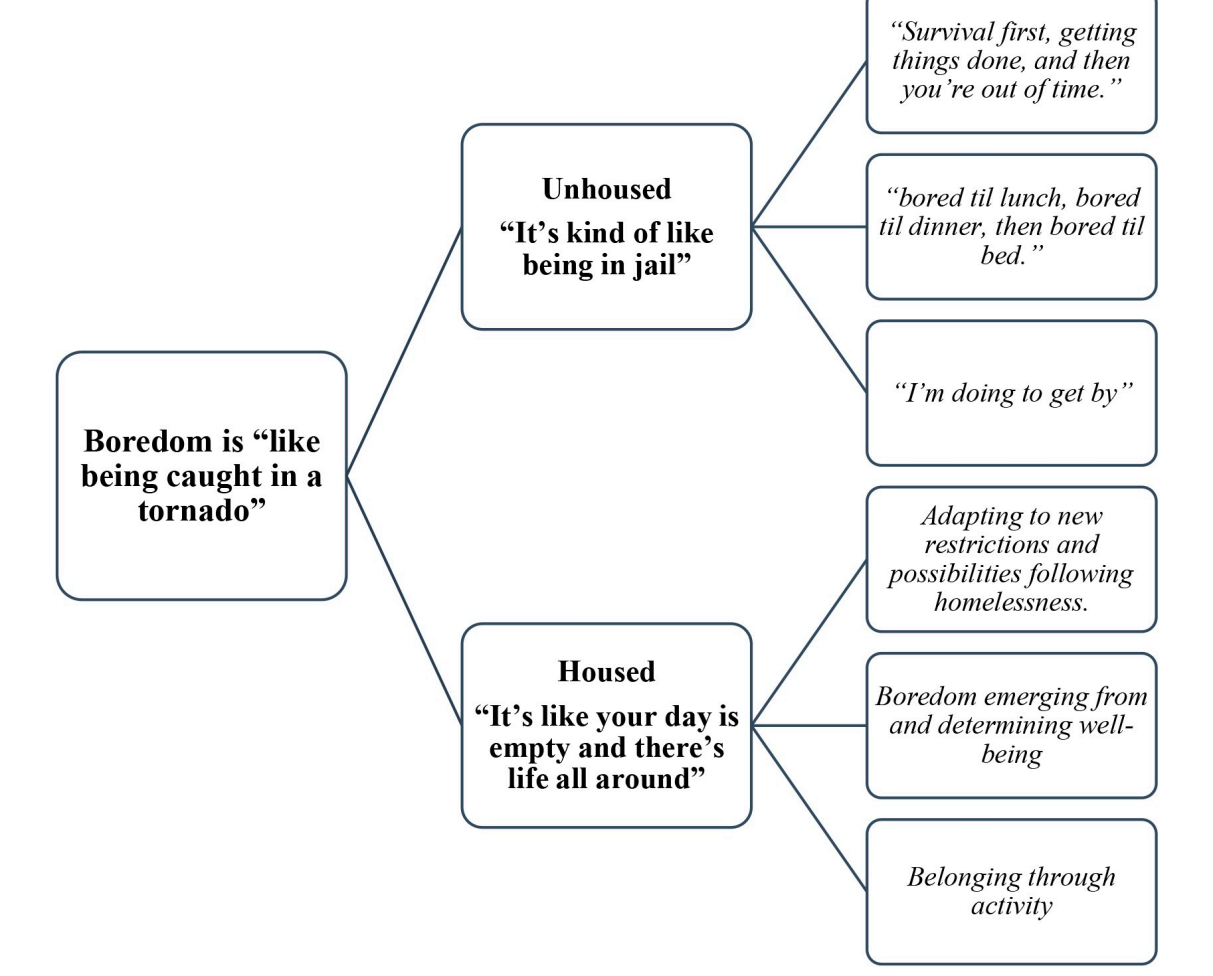
Qualitative theme structure.

We generated two ‘essences’ to describe participants’ experiences of boredom during and following homelessness. For unhoused participants, this essence was “*it’s kind of like being in jail*.” For housed participants, the essence was “*it’s like your day is empty and there’s life all around*.” A visual depiction of this theme structure is provided in Fig 1.

***During homelessness—“It’s kind of like being in jail”*.** Participants who were unhoused described how they were included within social networks in shelters and on the street, where they described being a part of a “*street family*” [Street Jesus, Unhoused]. While they were included in these social networks, they described how they were largely excluded from society: “*You know*, *you have to remember–you’ve been kicked out of the club*. *You’re not part of society anymore*” [Niel, Unhoused]. This profound social exclusion and having little money to occupy one’s time led to restrictions in opportunities for meaningful activity, leading to pervasive boredom: “*it costs too damn much to do anything…we’re bored*, *we’re sick*, *and we’re tired of it*” [Susan, Unhoused]. Rules imposed on their time use by shelters and other organizations that they accessed compounded this problem: “*In jail*, *there’s not a lot to do…I find it’s like being in jail*. *Like you gotta sign up for things*. *You gotta ask permission to do things*” [Jimmy Hat, Unhoused]. As a result, they spent their days either engaged in meeting their survival needs or “*just sitting around*” [Bud, Unhoused]. This essence was expressed through three themes generated in our analysis: “Survival first, getting things done, and then you’re out of time”; “bored til lunch, bored til dinner, then bored til bed”; and “I’m doing to get by”.

*“Survival first*, *getting things done*, *and then you’re out of time*.*”* Unhoused participants discussed at length how their time use was dictated by their survival needs, and how boredom emerged because their time use was taken up by activities that they didn’t choose to do but in which they needed to participate to survive:

*It feels like…there’s no time for anything*. *I’m not doing anything*, *though*, *so it is boredom*, *I guess…like nothing is going forward*, *and I can’t do anything about it…cause you gotta eat*. *Like you don’t have time to do any quality things ‘cause you still think*, *‘like where to get out of the cold’*, *or ‘what are you gonna eat*?*’* [Louise, Unhoused]

After completing survival activities, there was little choice in how participants could spend the time that was left given institutional, social, and financial constraints: “*there’s lots of things that I want to do*, *but can’t do*” [Louise, Unhoused]. They either spent this time engaged in activities that lacked meaning or did nothing at all: “*what we work on here is distraction*. *It’s not meaningful exercises…or meaningful endeavours*. *At least most of us…[are] just basically doing them to try and fill time*” [Niel, Unhoused].

*“Bored til lunch*, *bored til dinner*, *then bored til bed*.*”* Living in a state of survival and being situated in environments that limited engagement in meaningful activity led to boredom that lasted for much of the day. For many, it felt like the rule, rather than the exception: “*after breakfast is over*, *it’s bored til lunch*, *bored til dinner*, *then bored til bed*” [Shawn Foster, Unhoused]; “*I’m bored all the time*” [Apple, Unhoused]. For some, this felt like they had too much time: “*boredom*, *to me*, *feels like time is extended…[if] they had some way of stimulating your mind here*, *the time would go by faster*” [Niel, Unhoused]. For others, boredom felt like being trapped, like “*sitting in a jail cell*” [Peanut, Unhoused], or being “*sucked into the void…getting sucked into this all-encompassing*, *nothing-to-do*. *There’s no way to get out*” [Shawn Foster, Unhoused]. The boredom that participants described caused the days to meld together, and when they described this experience, it was as if they were suspended in a liminal space, sometimes due to the loss of important roles and relationships in their lives: “*I’m alone and I don’t have my kids…I feel like every day just blends*. *I had meaning before*” [Denver, Unhoused].

Participants described the impacts of this unrelenting boredom on their mental health, particularly. They frequently associated boredom with anxiety and depression: “*I’ll just be like sitting over there doing nothing*. *I’m seeing everybody come and go*, *and it’s like well*, *what am I doing*? *And I just get sad cause I’m not doing anything*” [Taylor, Unhoused]. This boredom led to a loss of motivation, hopelessness and a loss of purpose in their lives: “*So I feel like really I’m stuck*. *I’m in a hole*, *and I can’t get out of it*” [Louise, Unhoused]. Participants also discussed how having long periods of unoccupied time led to thinking deeply about their pasts, causing traumas to re-surface. Being alone with these thoughts with little opportunity for emotional processing gave rise to distress:

*You get flashbacks and stuff*, *right*? *You think about your past*, *and what you could’ve done*, *so you don’t end up here*…*You just got a lot of thinking time*, *I guess…that’s what I experience when I get bored*. *I just have a lot of thinking time*, *right*? *So I try not to be bored*. [Bud, Unhoused]

*“I’m doing to get by”*. Participants who were unhoused identified that they coped with the boredom that pervaded their lives by trying to find anything to do that might help them to escape the relentlessness of this experience. They felt a pressing “*need to just keep doing something*” [Niel, Unhoused]. They described constantly “*thinking of what to do next*, *like where to go next*” [Syres, Unhoused], and thinking “*a lot more about what I could do to bide the time away*” [Jimmy Hat, Unhoused]. Participants described how they coped with boredom primarily through the use of substances, which were widely available in shelters and on the street. When asked how she coped with boredom, Louise responded: “*I drink and drink as much as I can*” [Louise, Unhoused]. Speedy had a similar response and added: “*beer and weed actually speeds up the day*” [Speedy, Unhoused]. One participant indicated that when boredom was overwhelming:

*99% of the time*, *I find some good drugs…and get really fucking high for a day or two*, *and forget that you even exist…you roll yourself into getting as high as possible so you can stare at the sky*, *at the stars*, *and pretend you’re flying through them or something because life sucks…you need an escape*. [Shawn, Unhoused].

Participants also coped with the trauma that resurfaced during periods of boredom through substance use: “for me, I want to use mostly when I start thinking of bad things. I start thinking of my kids and I can’t stop those thoughts, right? So I need something to kind of distract” [Peanut, Unhoused].

***Following homelessness—“It’s like your day is empty and there’s life all around*.*”*** Participants who were housed following homelessness described how the boredom that they had experienced during homelessness persisted and became even more profound once they were housed. Boredom arose from a total lack of stimulation in their housing: “*the most noise I get is from pigeons on the balcony*” [Sandy, Housed]. The need to engage in survival activities lessened, and with few opportunities to engage in other activities, boredom often deepened: “*it’s hard to sit at home all day and do nothing*. *Like I don’t do anything*” [Rogo, Housed]. This led to feeling deeply disconnected: “*I become very numb and kind of forget that I’m even there or a person*. *Like literally*, *I forget I’m existing*” [April, Housed]. This profound boredom was associated with a sense of hopelessness and came as a surprise to participants as they hoped their transition to housing would be easier:

*There’s nothing to look forward to*. *It’s like your days are empty and yet there’s life all around*. *You don’t want to do anything*. *Like*, *my apartment is so quiet…and there’s people*. *Angry yelling*, *talking to themselves*, *around*. *Music’s playing*, *blasting usually*. *In all directions…Because my place is so quiet*, *it feels like I’m the only one who’s experienced such hollowness…this is the first time I’m on my own…as nice as it is*, *it’s very boring*. [Sandy, Housed]

This essence was expressed through three themes generated in our analysis: adapting to new possibilities and restrictions following homelessness; boredom emerging from and determining well-being; and belonging through activity.

*Adapting to new restrictions and possibilities following homelessness*. Participants described experiencing a lack of personal agency in how they could spend their time following homelessness. This lack of agency arose from the presence of ongoing poverty, poor social integration, and stigma. Living on a limited income was a serious barrier to engaging in their communities, which contributed to the boredom that they experienced: “*Having no money… It’s not having money…we’re really prevented from doing anything*, *so we sit around bored*” [Andrew, Housed].

For individuals living with mental illness, disclosure of their mental health condition became a difficult and calculated decision when trying to build new social networks that would provide access to meaningful activities after securing housing: “*I got a mental illness*, *but you don’t want too many people to know…and when you say that word*…*people will look at you a little different*. *Even if it’s just a little bit*” [Rogo, Housed]. Others felt that their ability to participate in activities in the community was influenced by feelings of safety in their new neighbourhoods: “*for me*, *personally*, *I have to take into account where I’m going*, *what I am doing*. *It’s like*, *is this place gonna be a safe place for me as a gay person*?” [Andrew, Housed].

Having stable housing enabled participants to think about their plans for the future, and many discussed the desire to return to work and school to increase their incomes and also to avoid boredom: “*at least I know that 10–12 hours of my day during the week I’m not bored*” [Nick Wilson, Housed]. Many faced barriers to returning to work and school, however, and discussed the need for accessible opportunities that accounted for their histories of homelessness and substance use. John described how he was engaged in a volunteer position that he wanted but had to leave because it was too difficult to manage so early after the transition to housing “*Somehow*, *they got me doing like an eight-hour day*. *And I was just up early in the morning and it was just…too much out of nowhere…once you spend your days drunk*, *you can’t just go back to one day waking up at five o’clock in the morning*” [John, Housed].

*Boredom emerging from and determining well-being*. Participants indicated that boredom pervaded their lives following homelessness and that disabilities associated with health conditions influenced the extent to which they experienced boredom. Mostly, participants described deleterious impacts of boredom on their well-being: “*I’ve spent a whole day just playing solitaire on my bed with the cards…I’ve sat there for literally eight hours and done it…I felt like I’m here wasting*” [Grandpa, Housed]. Participants also discussed how the presence of boredom led to the re-emergence of traumatic memories that caused distress: “*it plays on my head*. *It plays on my thoughts…too much time just sitting and doing nothing*” [Mia, Housed]. Some participants, however, recognized that boredom simultaneously imposed both positive and negative influences: “*I actually don’t mind a second of boredom*, *because then I can think creatively about what I’m going to do for the next little while to not be bored*, *or what I need to accomplish…but boredom*, *I think…can get you in trouble*” [Jude, Housed].

Similar to participants who were unhoused, housed participants described how the presence of boredom led to a desire to use substances for stimulation when opportunities for engagement in meaningful activity were limited: “*I’ve engaged in some drug use to deal with boredom*, *like crystal methamphetamine usage*. *Yeah*. *That’s a consequence of boredom…it’s just feeling fun*. *Feelings of pleasure*. *Normal everyday activities are just interesting to do*” [Andrew, Housed]. Participants who were housed also associated the experience of boredom with hopelessness and distress: “*it plays on my head*. *It plays on my thoughts*, *and it can be too much time just sitting doing nothing… Nothing*. *And not doing anything meaningful…it doesn’t feel good when there’s nothing*” [Mia, Housed].

While boredom influenced well-being, it also resulted from activity limitations caused by the presence of health conditions. For one participant living with physical disabilities, when asked if she experienced boredom, she responded:

*All day*, *everyday*. *Even at night when I’m awake…There’s not very much to do…and it’s hot*. *You know*, *you can’t go for walks because I have asthma…and I have to have a double knee replacement*, *so I’m in pain all the time*. *I can’t even walk*. *It’s just so hot that I would have to use my puffer constantly…to even go a couple of blocks*. [Grams, Housed]

*Belonging through activity*. Participants described how they experienced deep degrees of loneliness following homelessness and struggled to engage in their communities, leading to the emergence of boredom: “*I don’t know anybody here*, *so it’s just me by myself*. *And of course*, *I’m experiencing boredom*. *I just go walking and come back home*” [Max, Housed]. Being integrated in their communities was seen as critically important by participants: “*Without some form of community*, *you disappear*” [Andrew, Housed]. They saw meaningful activity, however, as an opportunity to belong in their communities, and some made active attempts to build on their social networks following homelessness. Sandy described how she had begun to serve coffee and tea to her neighbours as a way of mitigating boredom and connecting with others in her building:

*I go out and buy coffee*, *and Coffee Mate*, *sugar*, *things I need for serving coffee*, *and I serve coffee from 9*:*30 at night ‘til 1*:*00 in the morning*. *Just like a bar*. *And people are loving it*. *I’ve got a lot of customers*…*and they told me they really look forward to their coffee every night and their tea or whatever…one lady last night*, *she donated cookies and different things…and everybody gets together*, *and they talk…we have a lot of laughs*, *and we watch the news*. [Sandy, Housed]

## Discussion

We conducted this study to identify experiences of boredom, meaningful activity engagement, and their associations with key indices of psychosocial well-being during and following homelessness. Our findings build on published literature and provide further evidence of the pervasiveness of boredom in the lives of individuals during and following homelessness, and its serious, primarily detrimental, influence on psychosocial well-being [4, 6–8]. Beyond our pilot study, which included only two participants [5], this is the only known study which has focused on boredom in the transition to housing following homelessness. The findings of this study not only validate the findings of previous research but build on existing evidence by presenting an analysis representing a much larger sample across a broader range of urban contexts. This research furthers a growing body of literature which emphasizes the importance of meaningful activity in the lives of individuals during and following homelessness [47–53], and can be used to inform policy and practice aimed at addressing barriers to participation in meaningful activity to mitigate the deep degrees of boredom often experienced by individuals during homelessness and after securing a tenancy.

Our findings demonstrate the intimate relationship between boredom and psychosocial well-being among persons who experience homelessness. Participants in this study reported significantly higher boredom and lower engagement in meaningful activity than other populations, and poorer psychosocial well-being overall. In this study, boredom was associated with increased drug use, increased hopelessness, and lowered mental well-being, while conversely, engagement in meaningful activity was associated with lowered drug use, and increased mental well-being. These findings were consistent with qualitative interviews, both during and following homelessness, where participants emphasized the deleterious influence that boredom imposed upon their mental health. Further, participants described how substances enabled them to cope with the presence of boredom, and the trauma that re-surfaced during long periods of unoccupied time. These are important findings that provide a unique glimpse into the dynamics of substance use for persons who experience homelessness and explain one of the many contributors to the poor mental health typically observed in this population [35, 54]. While it is unlikely that engagement in meaningful activity will address all of the mental health and substance use challenges experienced by individuals during and following homelessness, our findings suggest that mitigating boredom may help to address some of these challenges.

A surprising and novel finding of this research was how boredom and meaningful activity were experienced following homelessness. Our quantitative analyses demonstrated that there were no differences in housed or unhoused participants on reported boredom or meaningful activity, and that the number of months of being housed, or unhoused in the past year did not predict boredom or meaningful activity engagement. Further, there were no statistically significant differences in these groups on other indices of psychosocial well-being. This suggests that meaningful activity engagement and boredom continue following homelessness, and that the psychosocial well-being of individuals leaving homelessness may not improve upon obtaining housing alone. Our qualitative analyses substantiated and contextualized these findings, with housed participants describing similar challenges with boredom and meaningful activity engagement as persons who were unhoused. Restrictions in meaningful activity engagement occurred for some of the same reasons for unhoused and housed participants and included ongoing poverty and social exclusion; however, different challenges emerged for participants who were housed. While having stable housing changed participants’ routines and provided them with a foundation on which they could participate in more meaningful activities, having limited social networks, living in ongoing poverty and being socially isolated following homelessness limited possibilities for meaningful engagement and resulted in boredom that was just as, or more profound, as when they were unhoused. This qualitative evidence, combined with the fact that our quantitative data illustrated no differences between unhoused and housed groups is a critically important finding, particularly with regard to homelessness prevention. If individuals have access to deeply affordable housing, *and* are experiencing wellness following homelessness, including having opportunities to participate in activities that are meaningful, it can be argued that such a person will be more likely to sustain their tenancy in the long term. While this study demonstrates the importance of meaningful activity engagement following homelessness, more research is needed to determine whether meaningful activity engagement can predict tenancy sustainment.

### Research and practice implications

For good reason, a large majority of research and practice in the area of homelessness is focused primarily on tenancy sustainment. While this is a critical goal, the findings of this study challenge the common assumption that housing alone necessarily leads to improvements in psychosocial well-being. This study emphasizes the need for greater research and practice attention on a broader range of indices of psychosocial well-being following homelessness that include meaningful time use. Research findings and practice evidence have given rise to a growing mass of researchers and practitioners who are calling for targeting a broader range of outcomes following homelessness, and a focus on targeting and measuring indices of thriving rather than simply sustaining a tenancy [1, 55–58]. While practice resources emphasize the inclusion of meaningful time use [59, 60], this outcome is rarely measured in the evaluation of interventions in research and practice. Measures of thriving following homelessness that include attention to boredom and meaningful activity engagement are needed, and are currently in development [61]. The availability of these measures will support researchers and service providers to attend to boredom and meaningful activity in their research and practice and will add to the growing body of evidence in this area.

Future research aimed at developing a more thorough understanding of the relationship between boredom, trauma and substance use is needed. Homelessness is recognized as a traumatic experience and persons who experience homelessness are known to experience high degrees of trauma both before and during homelessness [62, 63]. Trauma is also broadly recognized as a strong predictor of substance use [64]. Participants in this study described how the presence of pervasive boredom evoked the need to use substances simply to feel a sense of stimulation that was lacking in their lives, and to cope with the trauma that resurfaced during periods of unoccupied time. While we did not measure trauma quantitatively, participants associated boredom with trauma consistently across qualitative interviews. Future research focused on the relationship between boredom and substance use among individuals who experience homelessness is needed to uncover the ways in which boredom, trauma and substance use are related. Such research can inform the development and evaluation of practice and policy aimed at supporting persons who use substances and experience homelessness. Incorporating an understanding of how boredom and meaningful activity engagement factor into the substance use patterns of persons who experience homelessness is a novel contribution, and critically important given the high rates of substance use and trauma in this population [35, 54].

Overall, our findings suggest that interventions aimed at engaging individuals in meaningful activity are a serious gap in existing services. In a recent systematic review focused on the effectiveness of interventions for engaging individuals experiencing homelessness in meaningful activity, only nine moderate-high quality studies were identified [65]. The dire lack of intervention research in this area combined with the findings of this study suggest that future research aimed at developing and evaluating such interventions is needed. Participatory research that incorporates the perspectives of persons with experiences of homelessness, service providers and researchers may be an important strategy for co-designing and evaluating interventions designed to increase participation in meaningful activity.

Finally, persons with histories of homelessness are known to experience a high prevalence of mental illness [54], substance use disorder [35], and brain injuries [66]. These conditions are associated with the presence of cognitive challenges that may make boredom more likely irrespective of opportunities for engagement in meaningful activity (i.e. trait boredom). In this study, only a quantitative measure of state boredom was used (MSBS-8). Future research should incorporate measures of trait boredom that can help to identify whether boredom is related more or less to individual factors or the environmental contexts in which persons who experience homelessness are situated. This information will be critically important for informing the development of strategies aimed at mitigating the pervasiveness of this experience.

### Policy implications

Policymakers may consider the importance of meaningful activity engagement in evaluating and funding programs designed to support individuals who experience homelessness. The relentlessness of boredom in the lives of persons who are unhoused and housed following homelessness represents a serious social justice issue that has been associated with indices of well-being in this study and previous research [4–8]. Most of the participants in this study were receiving income support in the form of general and disability-related social assistance. Repeatedly, participants indicated that the income received from these programs is insufficient for meeting their daily needs, and prevented them from participating in meaningful activity, which ultimately gave rise to experiences of pervasive boredom. Social assistance rates in the province in which participants were recruited have been criticized for being insufficient for most [67]. Policymakers may consider increasing social assistance rates both to prevent homelessness, and to enable individuals living in low income to pay for their basic needs, which includes accessing meaningful activity. For some, this will mean increasing participation in employment. Only 15.2% of participants in the current study were employed. Previous research indicates that a large majority of persons who experience homelessness are unemployed, despite expressing the desire to work [68]. Services are needed to increase the accessibility of employment programs for persons with histories of homelessness that account for the high prevalence of disabilities that they experience. Finally, policymakers should be aware that the meaning of activities is highly subjective and phenomenological. As such, policies that are developed, and programs that are funded to increase participation in meaningful activities for persons who experience homelessness need to offer choice for this population in how their time is occupied.

### Limitations

The findings of this research represent the experiences of individuals during and following homelessness in three predominantly English-speaking, urban contexts in a high-income country. Further, the participants in both unhoused and housed groups in this study were primarily White and heterosexual. Transferring or generalizing the findings of this study to racialized, non-heterosexual groups should account for how these underrepresented groups may experience boredom, meaningful activity, and homelessness differently. Further, while the reliability of most of the standardized measures used in this study was satisfactory, internal consistency for the CIS subscales were in the ‘questionable’ range [69], and this should be accounted for when interpreting quantitative findings related to community integration in this study. Finally, it should be noted that the quotes provided to describe our themes in this article represent only a small fraction of the many quotes from interviews that could have been used to provide evidence of the themes highlighted in our findings. This should be acknowledged in any interpretation of the findings of this paper.

## Conclusions

Research pertaining to persons with experiences of homelessness has primarily focused on what is needed to support the security and maintenance of a tenancy and less on targeting thriving following homelessness. The findings of this research suggest that boredom and meaningful activity are important outcomes that should be accounted for in future research, practice, and policy related to supporting individuals following homelessness. The findings of this study suggest that this is not an outcome that is achieved through securing housing alone. Identifying existing and novel intervention strategies designed to increase participation in meaningful activities is an essential goal given the findings of this study. Future research designed to identify the nature of boredom (i.e. state vs. trait boredom) experienced by persons with histories of homelessness is needed to inform the development of these approaches. Finding ways to address boredom following homelessness is likely to promote thriving and may contribute to existing strategies for health promotion and homelessness prevention.
